# Toll-like receptor 2 expression is decreased on alveolar macrophages in cigarette smokers and COPD patients

**DOI:** 10.1186/1465-9921-6-68

**Published:** 2005-07-08

**Authors:** Daniel Droemann, Torsten Goldmann, Thorsten Tiedje, Peter Zabel, Klaus Dalhoff, Bernhard Schaaf

**Affiliations:** 1 Medical Clinic, Research Center Borstel, 23845 Borstel, Germany; 2 Clinical and Experimental Pathology, Research Center Borstel, 23845 Borstel, Germany; 3 Medical Clinic III, University of Lübeck, 23538 Lübeck, Germany

## Abstract

**Backround:**

Cigarette smoke exposure including biologically active lipopolysaccharide (LPS) in the particulate phase of cigarette smoke induces activation of alveolar macrophages (AM) and alveolar epithelial cells leading to production of inflammatory mediators. This represents a crucial mechanism in the pathogenesis of chronic obstructive pulmonary disease (COPD). Respiratory pathogens are a major cause of exacerbations leading to recurrent cycles of injury and repair. The interaction between pathogen-associated molecular patterns and the host is mediated by pattern recognition receptors (PRR's). In the present study we characterized the expression of Toll-like receptor (TLR)- 2, TLR4 and CD14 on human AM compared to autologous monocytes obtained from patients with COPD, healthy smokers and non-smokers.

**Methods:**

The study population consisted of 14 COPD patients without evidence for acute exacerbation, 10 healthy smokers and 17 healthy non-smokers stratified according to age. The expression of TLR2, TLR4 and CD14 surface molecules on human AM compared to autologous monocytes was assessed *ex vivo *using FACS analysis. *In situ *hybridization was performed on bronchoalveolar lavage (BAL) cells by application of the new developed HOPE-fixative.

**Results:**

The expression of TLR2, TLR4 and CD14 on AM from COPD patients, smokers and non-smokers was reduced as compared to autologous monocytes. Comparing AM we detected a reduced expression of TLR2 in COPD patients and smokers. In addition TLR2 mRNA and protein expression was increased after LPS stimulation on non-smokers AM in contrast to smokers and COPD patients.

**Conclusion:**

Our data suggest a smoke related change in the phenotype of AM's and the cellular response to microbial stimulation which may be associated with impairment of host defenses in the lower respiratory tract.

## Backround

COPD patients appear to have underlying pathologic abnormalities which facilitate bacterial colonisation and result in an increased rate of respiratory infections. Bacteria are detected in 40–60 % of exacerbations [1], and the significance of exacerbations for clinical course and decline of lung function is increasingly acknowledged [2]. The mechanisms of the increased susceptibility to bacterial infections are poorly understood. In addition to the impaired mucociliary clearance, deficient functions of the innate immune system seem to be of importance [3]. Incomplete elimination of bacterial pathogens contributes to continuing activation of immune effector mechanisms [4], possibly resulting in damage of the mucosa and parenchyma.

Tobacco smoking is known to induce inflammatory processes. Recent data demonstrated high concentrations of lipopolysaccharide (LPS) in cigarette tobacco as well as biologically active LPS in the particulate phase of cigarette smoke, suggesting a clinical relevance 5. AM play an orchestrating role in the pulmonary immune response. Pathogen recognition receptors (PRR) which are expressed on the macrophage's surface mediate the interaction between conserved patterns on microorganisms, pathogen associated molecular patterns (PAMP's), and host cells [6]. TLR- 4 together with CD14 and the MD2 adapter molecule serves as receptor for components from Gram-negative bacteria such as LPS. TLR2 predominantly recognizes components from Gram-positive bacteria such as lipoteichoic acid (LTA) and peptidoglycan (PGN) [6].

A disturbed regulation of PRR on monocytes and AM may affect the recognition of bacterial pathogens and the intracellular signaling as well as resulting effector mechanisms. A change in the PRR expression on AM from COPD patients may therefore be involved in the process of continuing inflammation and bacterial colonization. In our study we asked whether chronic cigarette smoke exposure alters the expression of PRR's in human monocytes/AM *ex vivo*. The surface expression of TLR 2, TLR 4 and CD14 was phenotypically characterized on circulating monocytes and AM obtained by BAL in COPD patients, healthy smokers and non-smokers. In addition, the response to LPS stimulation was evaluated at mRNA and protein level.

## Methods

### Study design

The study population consisted of three groups: 14 COPD patients (11 male, 3 female, FEV1 % predicted: mean 58, range 35–78), 10 healthy smokers (5 male, 5 female, FEV1 % predicted: mean 103, range 92–120), 10 young (6 male, 4 female, FEV1 % predicted: mean 108, range 98–118) and 7 elderly healthy non-smokers (6 male 1 female, FEV1 % predicted: mean 120, range 113–142). In the non-smoker group two age groups were recruited to exclude an age specific effect of PRR expression (young [A], elderly [B]).

Bronchopulmonary infection was excluded by clinical examination, systemic inflammatory markers and chest x-ray. The demographic data of the study population are summarized in table 1. This study was approved by the ethical committee of the University of Lübeck.

**Table 1:** Demographic data of the study population. Data are given as mean ± SD. AM = alveolar macrophages, Ly = lymphocytes, PMN = polymorphonuclear neutrophils.

	COPD (n = 14)	Smoker (n = 10)	Non-smoker	
			old (n = 7)	Young (n = 10)
Age	64.4 ± 9.2	30 ± 4.5	57.8 ± 6	26.8 ± 2.1
GOLD stage	II (n = 9) III (n = 5)			
Pack years	43.6 ± 13.8^#^	16.2 ± 5.2	0	0
Cell concentration BAL × 10^6^/100 ml	22.5 ± 10.8*	29.4 ± 19.0	13.0 ± 3.4	10.7 ± 5.1
Diff. Count BAL × 10^6^/100 ml AM	20.1 ± 9.7*	27.7 ± 18.2	11.1 ± 3.3	9.4 ± 4.8
Ly	1.2 ± 0.41	0.92 ± 0.31	1.6 ± 2.1	0.9 ± 0.29
PMN	0.95 ± 0.5^+^	0.66 ± 0.2	0.21 ± 0.23	0.21 ± 0.1
Gram stain smear	negative	negative	negative	Negative

^#^= p < 0.01 vs. smokers, * = p < 0.01 vs. non-smokers, ^+ ^= p < 0.01 vs. smokers and non-smokers.

### Bronchoscopy and isolation of BAL cells

After sedation with midazolam (3–10 mg) and local anesthesia with 2% lidocain bronchoscopically guided lavage was performed according to standard conditions in the middle lobe with instillation of 300 ml 0,9% NaCl. 20 ml aliquots were instilled and immediately reaspirated, recovery was 70–90 % for smokers and non-smokers and 35–68% for COPD patients. The lavage fluid was diluted to a final volume of 50 ml and filtered through four layers of gauze to eliminate remaining mucus [7]. Cells were differentiated counting a minimum of 600 cells on a cytocentrifuge smear (Cytospin II, Shandon, Frankfurt) stained with May-Grünwald/Giemsa solution. Gram-stains were performed on a cytocentrifuge smear, and culture for bacteria and yeast was routinely performed which did not show significant growth of pathogenic microorganisms. Viability was determined by trypan blue dye exclusion and the sample was diluted to a concentration of 10^6 ^viable cells/ml.

Studies of AM were always carried out in parallel with studies of peripheral blood monocytes from the same subject.

### Isolation of peripheral blood mononuclear cells (PBMC)

Peripheral venous blood was drawn 20–40 min. before bronchoscopy and PBMC were isolated from heparinized whole blood samples by Percoll density gradient centrifugation.

### Cell culture and in vitro stimulation

BAL cells and PBMCs were cultured in 6-well tissue plates (Nunc, Wiesbaden, Germany) using endotoxin-free RPMI 1640 medium (Biochrome, Berlin, Germany) supplemented with 2 mM L-glutamine (Gibco, Eggenstein, Germany) and 100 mg/ml streptomycin (Gibco, Eggenstein, Germany) at a density of 1 × 10^6 ^cells/ ml at 37°C in a 5% CO_2 _humidified atmosphere for a period of 4 h. Nonadherent cells were then carefully removed and the pellet was again cultured in medium which was supplemented with 1 μg/ml highly purified lipopolysaccharide (LPS, *Salmonella friedenau*, kindly provided by Prof. Brade, Research Center Borstel) for stimulation experiments. Preliminary experiments testing increasing LPS concentrations demonstrated a dose-dependent effect (TLR expression on AM in response to 0.1 μg/ml LPS: 12.8 rMFI vs. 16.1 rMFI after 1 μg LPS/ml, mean of n = 3 experiments).

### Flow cytometry

To facilitate flowcytometric analysis of the AM, we used a previously described quenching technique which reduces intracellular fluorescence and permits analysis of fluorochrome-labeled antibodies by flow cytometry [8]. After 4 h of *in vitro *cultivation cells were analyzed for surface antigen expression. The expression of TLR2, TLR4 and CD14 on AM and autologous monocytes was determined using a fluorescence activated cell sorter (FACS) Calibur (Becton Dickinson, Heidelberg, Germany). Data acquisition and analysis were performed with CellQuest software (Becton Dickinson, Heidelberg, Germany). Each measurement contained ≥ 20,000 cells in the AM and monocyte population determined by characteristic forward/orthogonal light scattering in a density plot and positive HLA-DR expression. For compensation of the autofluorescence of AM, cell preparations were performed using crystal violet like recommended previously [8]. For permeabilization Intraprep reagent was used (Beckman Coulter, Krefeld, Germany). Antibodies against the following epitopes were used. PE-labeled: TLR2, TLR4, isotype controls (eBioscience, San Diego, USA), CD14, isotype control; PE-CY-5-labeled: HLA-DR, isotype control (the latter all purchased from BD Pharmingen, Hamburg, Germany). The expression of surface markers was calculated as relative mean fluorescence intensity (rMFI = monoclonal antibody/ corresponding isotype control) since no bimodal distribution was found.

### In situ hybridization (ISH)

In a subgroup of 13 non-smokers and six COPD patients BAL cells were attached on SuperFrost Plus microscope slides (Menzel-Gläser, Braunschweig, Germany) by centrifugation for 5 minutes at 450 rpm at high acceleration in a Cytospin 2 centrifuge (Shandon, Frankfurt, Germany) and dried for 10 min at room temperature. After overnight fixation at 4°C in Hepes-Glutamic acid buffer mediated Organic solvent Protection Effect (HOPE) solution, cells were incubated with acetone/glyoxal for 1 hr. at 4°C, 6 times dehydrated with acetone for 30 min. at 4°C, followed by two incubations in isopropanol (10 min at 60°C, 2 min. at 60°C) and air dryed. Rehydration was achieved by incubation in 70% (vol/vol) acetone for 10 min. at 4°C and DEPC treated water for 10 min. at 4°C [9-11]. Slides were air dried. A TLR2 probe for ISH was prepared as previously described [12], and ISH was carried out overnight in moist chambers at 46°C. Post hybridization washes and the detection of hybrids have been described previously [9,12]. The generation of signals was achieved in approximately 10 minutes. Slides were mounted and digitally photographed.

### Statistics

Nonparametric statistics were used throughout the study. Data are given as mean ± SD, if not otherwise indicated. Surface antigen expression on AM and monocytes from COPD patients, smokers and non-smokers were tested by the analysis of variance followed by Kruskal Wallis test and the Wilcoxon signed rank test was used for comparison of paired samples (pulmonary vs. systemic cells from the same persons and stimulation experiments). A p value < 0.05 was considered as significant.

## Results

### Differential PRR surface pattern of monocytes and AM

**Figure 1:**
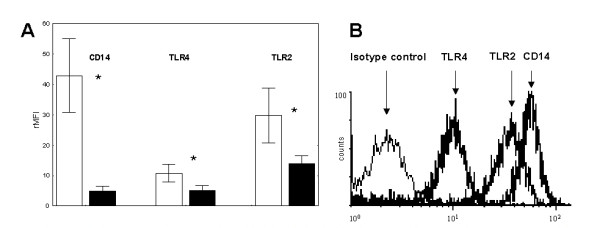
(A) Flow cytometry expression of CD14, TLR4 and TLR2 on monocytes and autologous alveolar macrophages (AM). rMFI (± SD) is shown from non-smokers (n = 10). White bars = monocytes, black bars = AM. * = p < 0.01 vs. AM. rMFI = relative mean fluorescence intensity. (B) Representative histogram from experiments with monocytes.

The expression of CD14, TLR4 and TLR2 was higher on monocytes compared to AM in non-smokers (A and B), smokers and COPD patients (CD14: 42.92 ± 12.15 vs. 5 ± 1.56; 36.1 ± 15.4 vs. 4.7 ± 1.6 [nonsmoker groups], 32.4 ± 16.2 vs. 4.09 ± 0.69 and 40.8 ± 10.7 vs. 4.3 ± 1.53 relative mean fluorescence intensity (rMFI), p < 0.01; TLR4: 10.81 ± 2.88 vs. 5.19 ± 1.58; 8.7 ± 5.2 vs. 5.3 ± 2.1; 12.3 ± 4.6 vs. 4.28 ± 0.7 and 9.2 ± 3.9 vs. 4.62 ± 1.38 rMFI, p < 0.01; TLR2: 29.71 ± 9.01 vs. 13.98 ± 2.54; 24.8 ± 10.6 vs. 12.07 ± 3.5; 32.3 ± 8.4 vs. 6.59 ± 1.42 and 27.3 ± 10.8 vs. 6.08 ± 1.5 rMFI, p < 0.01). There was no significant difference in the PRR expression on monocytes from non-smokers (A and B), smokers, COPD patients. Figure 1a shows data from non-smokers (A), a representative histogram demonstrates the ratio of isotype control to surface molecule fluorescence (figure 1b). In addition there was also a difference in the percentage of positive cells (monocytes vs. AM, CD14: 96 vs. 9.5; TLR4: 69 vs. 11, TLR2: 87.5 vs. 22.5 % positive).

### PRR expression of AM from non-smokers, smokers, COPD patients

**Figure 2:**
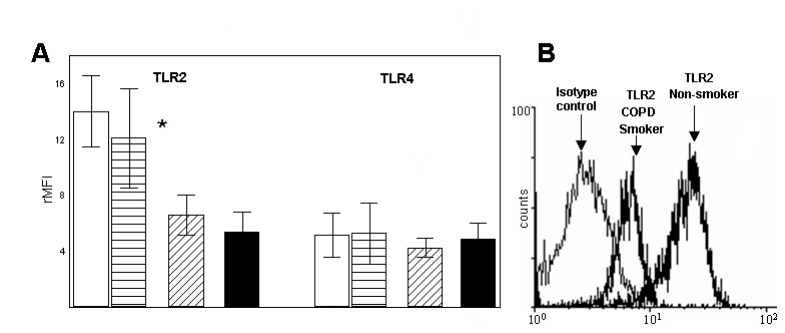
(A) Flow cytometry expression of TLR2 and TLR4 on alveolar macrophages (AM). rMFI (± SD) is shown from non-smokers (white bars [young], n = 10, horizontal hatched bars [elderly], n = 7), smokers (diagonal hatched bars, n = 10) and COPD patients (black bars, n = 14). * = p < 0.01 vs. smokers and COPD patients AM. rMFI = relative mean fluorescence intensity. (B) Representative histogram from experiments with AM.

Comparing AM from non-smokers (A and B), smokers and COPD patients we detected a markedly lower expression of TLR2 on AM from smokers and COPD patients (13.98 ± 2.54 and 12.07 ± 3.5 [nonsmoker groups] vs. 6.59 ± 1.42 and 6.08 ± 1.5 rMFI, respectively, p < 0.01) (figure 2a). There was no difference in the expression of CD14 and TLR4 between the groups (CD14: 5 ± 1.56 and 4,7 ± 1,6 vs. 4.09 ± 0.69 and 4.3 ± 1.53 rMFI; TLR4: 5.19 ± 1.58 and 5,3 ± 2,1 vs. 4.28 ± 0.7 and 4.62 ± 1.38 rMFI, respectively). Percentage of positive cells showed analogous data (nonsmokers (A and B) vs. smokers, COPD patients, TLR2: 22.5 and 19.2 vs. 12.9 and 12.2; CD14: 9.5 and 8.5 vs. 8.1 and 8.7; TLR4: 11 and 12.5 vs. 8.9 and 10.3 % positive). A representative histogram demonstrates the ratio of isotype control to surface molecule fluorescence (figure 2b).

### Regulation of PRR expression on AM

**Figure 3:**
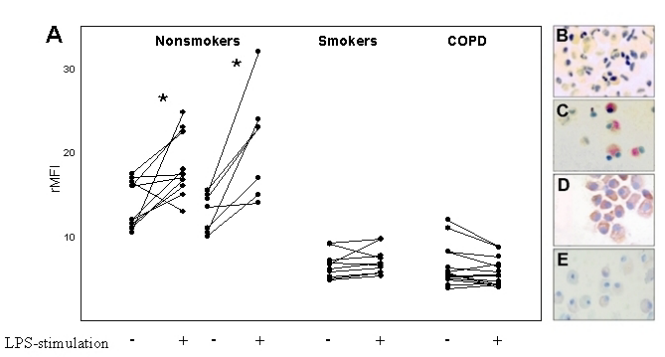
LPS-stimulation of TLR2 protein and mRNA on alveolar macrophages (AM). (A) Flow cytometry expression. rMFI (± SD) is shown from non-smokers (young n = 10, elderly n = 7), smokers (n = 10) and COPD patients (n = 14). rMFI = relative mean fluorescence intensity. * = p < 0,04. *In situ *hybridization targeting TLR2 mRNA of HOPE-fixated BAL cells. Cells from non-smokers before (B) and after (C [young], D [elderly]) LPS-stimulation. Cells from COPD patients (E) after LPS-stimulation (Anti-DIG-AP-New-fuchsine; 600x).

To study the regulation of PRR expression after ligand stimulation, cells were exposed to LPS (1 μg/ml) which led to an increased expression of TLR2 on AM from non-smokers (18.35 ± 4.24 vs. 13.98 ± 2.54 rMFI [A] p < 0.04; 21.1 ± 6.23 vs. 12.07 ± 3.5 [B] p < 0.04). In contrast, cells of smokers and COPD patients did not respond to LPS stimulation with increased TLR2 expression (7.09 ± 1.42 vs. 6.59 ± 1.42 rMFI [smokers], p = n.s.; 6.63 ± 2.40 vs. 6.24 ± 1.71 rMFI, [COPD patients], p = n.s.) (figure 3a). Percentage of positive cells showed analogous data (nonsmokers: 28 vs. 22.5 [A], 30.1 vs. 19.2 [B], smokers: 13.8 vs. 12.9, COPD patients: 13.0 vs. 12.2 % positive). There was no effect of LPS stimulation on the surface expression of CD14 and TLR4 in all groups. Representative results of ISH targeting human TLR2-mRNA before and after LPS-stimulation in non-smokers (A and B) and COPD patients are photographically displayed in figure 3b–e. HOPE-fixed specimens showed a good preservation of morphology after the ISH procedure. The generation of signals was achieved in approximately 10 minutes. Strong signals were found in AM of non-smokers (A and B) after LPS stimulation. Nonspecific signals were not detected in control preparations, in which specific DNA probes were substituted by hybridization buffer alone or an irrelevant probe.

## Discussion

In this study we comparatively evaluated the influence of chronic smoke exposure on the pattern of TLR2, TLR4 and CD14 expression in human AM and monocytes in COPD patients, smokers and non-smokers. We observed a significantly decreased expression of PRR's on AM compared to monocytes. The main finding was that AM from COPD patients and smokers show an equally decreased surface expression of TLR2 compared to non-smokers of two age groups. In addition, an upregulation of TLR2 after LPS stimulation was only observed on non-smokers AM.

An increased TLR2 surface expression on human monocytes in response to LPS-stimulation has been described previously [13], whereas on the transcriptional level divergent data have been reported showing either upregulation [14,15], or downregulation [16,17] of TLR2-mRNA depending on timing and dose of stimulation. In addition, differential regulation of TLR2 by IL-1, IL-10 and GM-CSF (upregulation) and IFN-gamma, TNF-α and IL-4 (downregulation) has been observed [13]. Therefore, the net result of stimulation *in vivo *will depend on the local balance of inflammatory mediators. What are the possible consequences of LPS-stimulated TLR2 expression? Whole gram-negative bacteria are recognized not only through TLR4 (by LPS) but also TLR 2 (by bacterial lipopeptides). In this setting upregulation of TLR2 which occurs mainly with high LPS doses, may provide an additional mechanism to sensitize cells against large microbial challenges. Moreover, TLR2 is the main PRR recognizing gram-positive bacteria, and recent studies have shown that TLR2 -deficient animals are at high risk for succumbing to invasive pneumococcal infections [18]. Thus, the blunted TLR2 expression of AM from smokers and COPD patients after LPS stimulation may impair antimicrobial defenses in the lower respiratory tract. Recently a reduced TLR expression in aging mice was demonstrated [19]. However this factor seems to be without influence on our results since comparable levels of PRR expression on monocytes and AM from healthy young and elderly non-smokers were observed.

The mechanisms of the smoking induced alteration of pulmonary immune functions are poorly understood. On the one hand smoking is known to induce inflammatory processes by activating AM and epithelial cells leading to production of TNF-α, IL-8 and LTB4 and subsequent neutrophil recruitment [20]. Accordingly, in BALF and sputum of patients with COPD neutrophil activation and elevated levels of proinflammatory cytokines and chemokines have been found [21]. On the other hand a depressed capacity for LPS-induced cytokine release of TNF-α and IL-6 from AM of smokers was described [22]. Skold and colleagues found a higher expression of CD11a, CD54 and CD71 in non-smoker's AM compared with smokers [23]. CD11a (LFA-1) and its ligand play an important role in the interaction between antigen presenting cells and T-lymphocytes. In addition the metabolic response after *in vitro *stimulation with phorbol myristate acetate (PMA) was higher in non-smokers than in smokers AM. Dandrea *et al*. observed a reduced inflammatory cytokine release in cultured AM from smokers in response to LPS by simultaneous exposure to NO2 compared to non-smokers [24]. Cultured human bronchial epithelial cells from COPD patients release lower levels of inflammatory mediators such as TNF-α and IL-8 than similar preparations from non-smokers or smokers without COPD, suggesting that downregulation of inflammatory mediator release may also occur in bronchial epithelial cells of individuals with COPD [25].

Our data demonstrating an altered AM phenotype with reduced expression of TLR2 in smokers and COPD patients suggest that a continuous exposure to microbial products in this disease provided by bacterial colonization and LPS present in tobacco smoke [5] may downmodulate the pulmonary immune response. Whether this is due to the selection of a heterogenous macrophage subpopulation in the pulmonary compartment or to a general AM phenotype change under the environmental conditions described cannot be firmly differentiated from our data. However, with regard to the continous distribution of TLR expression intensities found by flow cytometry the latter possibility seems more likely. *In vitro *it was shown that the TLR response is downregulated after repetitive stimulation [26]. Interestingly a hyporesponsiveness of cells to the TLR-4 ligand LPS was shown as well after preincubation with ligands for TLR2 and vice versa, which indicates the existence of common signaling pathways of the TLR system [27]. It is tempting to speculate that this phenomenon also plays a role under conditions of chronic stimulation with bacterial components *in vivo *as suggested by the missing effect of LPS-stimulation on TLR2 expression on cells from smokers and COPD patients. This finding was confirmed using *in situ *hybridization targeting TLR2, which was recently demonstrated by our group on AM and alveolar epithelial cells type II in the human lung [12]. Although there was only a small overlap between non-smokers on the one hand and smokers and COPD patients on the other we observed a large variability in the extent of response to LPS stimulation in the non-smoking group which is a well-known phenomenon with regard to the release of biologic mediators as proinflammatory cytokines [28]. In contrast, no difference in TLR4 expression between smokers, non-smokers, COPD patients was observed in our study which may be due to the generally weaker expression of this molecule on monocytes and AM.

Functionally relevant polymorphisms of TLR's have been found in persons with endotoxin hyporesponsiveness (TLR-4) and staphylococcal sepsis (TLR-2) [29,30]. Data regarding their relevance in COPD are not available. The generally lower expression of PRR's on AM's compared to monocytes is comparable to data reported for dendritic cells and may accompany the differentiation process of monocytes to macrophage populations [15]. Regarding the effect of chronic smoke exposure on blood cells we did not find any difference in the PRR expression on monocytes between smokers and non-smokers (data not shown) suggesting a compartmentalized effect of tobacco smoking. In contrast Lauener et al. demonstrated a significantly increased expression of CD14 and TLR2 on blood cells of farmers' children compared to non-farmers' children [31].

In conclusion, the altered phenotype of smokers AM could play a role in decreased cellular responses to microbial stimulation facilitating persistent infection. Further studies regarding to the functional relevance of these findings and their contribution to the pathogenesis of COPD could lead to more effective treatment regimens of this disease.

## Abbreviations

AM = alveolar macrophage; BAL = bronchoalveolar lavage; COPD = chronic obstructive pulmonary disease; FACS = Fluorescence activated cell sorter; GM-CSF = granulocyte-macrophage colony-stimulating factor; HOPE = Hepes-Glutamic acid buffer mediated Organic solvent Protection Effect; IL = interleukin; IFN-γ = interferon-γ; LTA = lipoteichoic acid; LPS = lipopolysaccharide; PBMC = peripheral blood mononuclear cell; PE = phycoerythrin; PGN = peptidoglycan; PMA = phorbol myristate acetate; PMN = polymorphonuclear neutrophils; PRR = pattern recognition receptor; rMFI = relative mean fluorescence intensity; TLR = Toll-like receptor; TNF-α = tumor necrosis factor-α

## Authors' contributions

DD carried out the flow cytometry and was involved in the design of the study and drafting the manuscript. TG performed the *in situ *hybridization and conceived of the study. TT carried out cell culture experiments and was involved in drafting the manuscript. PZ, KD and BS conducted the clinical part of the study and were involved in the design and coordination of the study. All authors read and approved the final manuscript.
